# P063: Tackling VRE in a community hospital with teamwork and tenacity: lessons learned

**DOI:** 10.1186/2047-2994-2-S1-P63

**Published:** 2013-06-20

**Authors:** P Raggiunti, S Vinod, S Vinod

**Affiliations:** 1 Infection Control, Rouge Valley Health System, Toronto, Canada

## Introduction

A VRE Outbreak was declared November 2010 in a large community hospital impacting two acute care and two complex continuing care units. A number of infection control measures were immediately put into action with an ongoing focus on environmental controls, adherence to hand hygiene and isolation protocols. Despite concerted efforts to resolve the VRE outbreak in a timely fashion, ongoing transmission of VRE continued with three distinct peaks identified throughout the 17 month period. A total of 110 patients became colonized with VRE.

## Objectives

Outbreak management

## Methods

An outbreak management team was established. Control measures focused on: hand hygiene, contact precautions, personal protective equipment, dedicated equipment, active surveillance, patient, staff and visitor control measures, assessment of furniture and equipment, education, audits, enhanced laboratory testing, cleaning and disinfection etc.

## Results

Three peaks were noted during the 17 month period. The VRE isolates were tested by PFGE to determine relatedness. The first peak was noted in Jan 2011, the cluster of isolates revealing to be type A, the second peak in April 2011 had a cluster of type A1 and the third peak followed in September 2011 with a cluster of type A14. This indicates clonal spread of organisms. Resurgence of the outbreak in April 2011 from what appeared to have been controlled, strongly supports the long-term survival of VRE in the environment from our experience. Environmental reservoirs such as furniture with porous surfaces including wooden or upholstered furniture, furniture with impaired integrity were reservoirs for VRE bacteria as evidenced by VRE positive environmental cultures.

## Conclusion

Shared equipment and shared assignments for staff appears to have spread the organism to different units reflecting indirect spread of VRE. Patient transfers from acute to continuing care contributed to the spread. Deeper scrutiny of the outbreak revealed the need to enhance processes to achieve good infection prevention control practices with a focus on relentless teamwork and ongoing communication which helped in mitigating the outbreak. It is very important to maintain the morale of staff, maintain transparency of the situation and have an ethical approach towards the management of patients on contact precautions to ensure the qualtiy of their care.

## Disclosure of interest

None declared

